# Letter and word identification in the fovea and parafovea

**DOI:** 10.3758/s13414-021-02273-6

**Published:** 2021-03-21

**Authors:** Michele Scaltritti, Jonathan Grainger, Stéphane Dufau

**Affiliations:** 1 Dipartimento di Psicologia e Scienze Cognitive, Università degli Studi di Trento, Corso Bettini 31, 38068 Rovereto, TN Italy; 2 Laboratoire de Psychologie Cognitive, Aix Marseille University and CNRS, Marseille, France; 3 Institute for Language Communication and the Brain, Aix-Marseille University, Marseille, France

**Keywords:** Reading, Word recognition, Parallel word processing, Parallel letter identification

## Abstract

We investigated the extent to which accuracy in word identification in foveal and parafoveal vision is determined by variations in the visibility of the component letters of words. To do so we measured word identification accuracy in displays of three three-letter words, one on fixation and the others to the left and right of the central word. We also measured accuracy in identifying the component letters of these words when presented at the same location in a context of three three-letter nonword sequences. In the word identification block, accuracy was highest for central targets and significantly greater for words to the right compared with words to the left. In the letter identification block, we found an extended W-shaped function across all nine letters, with greatest accuracy for the three central letters and for the first and last letter in the complete sequence. Further analyses revealed significant correlations between average letter identification per nonword position and word identification at the corresponding position. We conclude that letters are processed in parallel across a sequence of three three-letter words, hence enabling parallel word identification when letter identification accuracy is high enough.

## Introduction

Psycholinguistic models of reading have greatly benefited from investigations of visual word recognition (Balota, 1994), tackling the representations and processes involved in the identification of written words presented in isolation. Nonetheless, it is immediately clear that in most reading contexts, visual information from multiple words becomes simultaneously available to the reader. Whether the multiple words that are available to the reader, given visibility constraints (Grainger, Dufau, & Ziegler, 2016), are processed serially or in parallel is a hotly debated issue (e.g., Reichle, Liversedge, Pollatsek, & Rayner, 2009; Snell & Grainger, 2019). One model of eye movement and reading, the E-Z Reader model (Reichle et al., 2009; Reichle, Pollatsek, Fisher, & Rayner, 1998), favors a serial perspective in which words are identified one by one. In these models, parafoveal processing, i.e., the processing of stimuli next to the fixated one, which goes beyond purely visual pre-attentive processing, occurs when attention has been shifted to that location in preparation for an eye movement. However, evidence against a strictly serial approach to reading has been growing in recent years (see Snell & Grainger, 2019, for a summary of the evidence), and consequently a parallel processing framework has gained momentum in reading research (Engbert, Nuthmann, Richter, & Kliegl, 2005; Reilly & Radach, 2006; Snell, van Leipsig, Grainger, & Meeter, 2018). In particular, OB1-reader (Snell et al., 2018) is the first parallel processing model to actually implement letter and word-identification processes. In the present study, we test some specific predictions derived from OB1-reader, and notably the relation between letter identification and word-identification processes in foveal and parafoveal vision.

At a more general level of theorizing, current models of reading need to connect letter-identification processes with word-identification processes in foveal and parafoveal vision. To date, OB1-reader is the only computational model to have bridged this gap. In order to put this model to test, and to guide future modeling efforts in the same direction, we need precise knowledge about how well readers can identify letters and words in the fovea, and particularly when this information is obtained under similar testing conditions for both types of stimuli, with an aim to connect the two. The present study fills this gap.^1^

In the present work we combine, for the first time, estimates of word identification accuracy in multiple word displays as well as corresponding estimates of letter identification accuracy. Much prior research has investigated letter identification accuracy in multi-letter displays (see Tydgat & Grainger, 2009, for a review of the early research). This research has highlighted the role of two major determinants of letter identification: visual acuity and crowding. Visual acuity is maximal in the fovea and decreases as a function of eccentricity from fixation. Correspondingly, experimental paradigms that require participants to report the identity of a post-cued character from a briefly displayed string of letters have consistently revealed higher accuracy for the letter at fixation. Moving away from fixation, accuracy in letter identification becomes progressively lower, except for the initial and the final letters. Here, the diminished visual acuity is in fact compensated by a reduction of crowding interference because of the reduced number of flanking characters for letters in the first or final position (e.g., Marzouki & Grainger, 2014; Merikle, Coltheart, & Lowe, 1971; Merikle, Lowe, & Coltheart, 1971; Mewhort & Campbell, 1978; Stevens & Grainger, 2003; Tydgat & Grainger, 2009). Over and above these major driving forces in letter identification, researchers consistently reported a first-letter advantage (e.g., Johnson & Eisler, 2012; Scaltritti & Balota, 2013; Tydgat & Grainger, 2009). This has been explained as stemming from low-level perceptual adaptation of the receptive fields of letter detectors (e.g., Chanceaux & Grainger, 2012; Grainger, Tydgat, & Isselé, 2010; Tydgat & Grainger, 2009) and/or visuo-spatial attention (Aschenbrenner, Balota, Weigand, Scaltritti, & Besner, 2017; Scaltritti, Dufau, & Grainger, 2018).

More recent research has begun to investigate word identification in multi-word displays (Declerck, Wen, Snell, Meade, & Grainger, 2020; Snell & Grainger, 2017; Wen, Snell, & Grainger, 2019). Mimicking the post-cued letter-in-string procedure used in the studies described in the preceding paragraph, this research has used the Rapid Parallel Visual Presentation (RPVP) procedure, with brief (200-ms) simultaneous presentation of four words followed by a post-mask and post-cue to indicate the word in the sequence whose identity should be reported. Akin to the classic word superiority effect (Reicher, 1969; Wheeler, 1970), Snell and Grainger (2017) found superior word report when the target word was presented in a grammatically correct sequence compared with an ungrammatical sequence of the same words – a sentence superiority effect (see also Declerck et al., 2020; Massol & Grainger, 2020; Wen et al., 2019). Particularly relevant for the present study is that the serial position functions observed in each of these studies systematically revealed superior performance at position 2, with performance tending to drop linearly from that position, with worst performance at the first position except in the beginning readers tested by Massol and Grainger.

In the present study we measured word identification accuracy in three-word displays, as well as the accuracy in identifying the component letters of these words when presented at the same location in a context of nonword sequences. Specifically, in the word-identification task, participants were presented with three three-letter words for 117 ms followed by a backward mask accompanied by a post-cue to indicate which of the three words was to be identified. In the letter identification block, participants were presented with a sequence of three three-letter nonwords formed by shuffling the letters used in the word-identification task and had to identify one of the letters in that sequence. It is important to note that for each word at a given location in the word-identification experiment we tested the identification accuracy of each component letter at exactly the same location (same position of the nonword trigram among the three nonwords and same within-trigram position). With this experimental design, other than investigating the serial position function for word and letter identification in multiple arrays, we were thus also able to explore the relationship between letter and word identification in this context by assessing the extent to which the identification of letters at different positions correlates with accuracy in word identification.

One straightforward prediction of OB1-reader (Snell et al., 2018) is that letters are processed in parallel across multiple words and, in the absence of top-down constraints from sentence-level representations, it is the efficiency of such letter-identification processes that determines ease of identification of words in the parafovea. EZ-Reader on the other hand, assumes that only feature-level information can be processed in parallel across multiple words in the absence of a shift of attention (Angele, Tran, & Rayner, 2013). This approach therefore predicts that it is not actual letter identification that should critically determine how well words are identified in the parafovea. The present study puts these different predictions to test by examining the relation between letter-identification accuracy and word-identification accuracy in the fovea and parafovea.

## Method

### Participants

Twenty-two native French speakers (five males, 17 females; M_age_ = 22.95 years; SD_age_ = 4.70) took part in the experiment. All of them reported normal or corrected-to-normal vision. Participants provided written informed consent and were compensated with €10 for their participation. All the procedures obtained ethics approval from the Comité de Protection des Personnes SUD-EST IV (No. 17/051).

### Materials and design

#### Word-identification task

Ninety three-letter French words were selected from the Lexique3 database (New & Pallier, 2020; New, Pallier, Brysbaert, & Ferrand, 2004). In terms of their consonant(C)-vowel(V) structure, the majority had a CVC (65.56%) or a CVV (18.89%) structure. Other structures were more sporadic (CCV = 1.11%; VCC = 1.11%, VCV = 6.67%; VVC = 4.44%; VVV = 2.22%), reflecting the features of the French language. The 90 words were randomly partitioned into three subsets of 30 words each. The three subsets were matched for a series of psycholinguistic properties listed in Table 1. All the psycholinguistic variables were taken from the Lexique3 database.

**Table 1:** Mean values (SDs within parentheses) of psycholinguistic variables of the words used in the experiment

Variable	Subset 1	Subset 2	Subset 3
Frequency (log)	1.69 (1.15)	1.51 (1.08)	1.47 (1.18)
N. homographs	1.53 (0.63)	1.53 (0.86)	1.33 (0.71)
N. homophones	5.70 (4.07)	5.77 (3.02)	5.50 (3.36)
N. Phonemes	2.63 (0.49)	2.60 (0.62)	2.50 (0.57)
Orth. N.	11.77 (4.58)	12.87 (5.10)	11.40 (4.42)
Phon. N.	22.83 (8.08)	22.43 (7.72)	22.27 (6.77)
N. Syllables	1.07 (0.25)	1.03 (0.18)	1.03 (0.18)
OLD	1.23 (0.26)	1.18 (0.18)	1.20 (0.21)
PLD	1.07 (0.19)	1.04 (0.10)	1.05 (0.18)

*Orth. N.* orthographic neighborhood size, *Phon. N.* phonological neighborhood size, *OLD* orthographic Levenshtein distance, *PLD* phonological Levenshtein distance

Thirty triplets of words were then created, with each word in the triplet drawn from one of the three subsets. Each participant saw each triplet three times, with words of the triplet assembled in three different orders following a Latin-square design (yielding three unique combinations) so that (1) each position of each triplet was probed once within each participant, and (2) all words were probed only once within each given participant. The word probed at each position within each triplet was counterbalanced across participants.

There was thus one experimental factor, word position (1–3), manipulated within participants and within items.

#### Letter-identification task

For each combination of each triplet of words, nine nonword versions were created by pseudo-randomly scrambling the order of the nine letters included therein. Within each scrambled version, only one letter of one word preserved its original location and served as the target for the letter-identification task. None of the other letters appeared in the same position as in the original triplet. Across the nine scrambled exemplars, each one of the letters from each word retained its original position, and thus served as the target for the identification task. For any triplet of words seen in the word-identification task, each participant saw all the corresponding nine scrambled versions in the letter-identification task. Care was taken so that any nonword did not correspond to an actual word in French. No other constraint was imposed when creating the nonwords and, in particular, no adjustments were made in order to approach (or avoid) a word-like structure (see Appendix A for additional analyses). As a consequence, the resulting CV structures were quite randomly distributed across the different possibilities (CCC = 17.41%; CCV = 16.33%; CVC = 15.38%; CVV = 13.23%; VCC = 12.55%; VCV = 5.26%; VVC = 12.69%; VVV = 7.15%).

In the letter-identification task there were two experimental factors: nonword position (1–3) and letter-in-nonword position (1–3).

### Apparatus and procedure

The experimental procedure and data acquisitions were controlled by E-Prime 2 software (version 2.0.10.252, Schneider, Eschman, & Zuccolotto, 2002). Participants were seated in front of a computer screen running with a 2,050 × 1,440 resolution, at a distance of approximately 50 cm and with their head resting on a chin-rest. Stimuli appeared in black on a gray background (RGB = 210, 210, 210) in 16-pt Courier New lowercase font.^2^ Each string subtended 2.9° of visual angle.

**Fig. 1:**
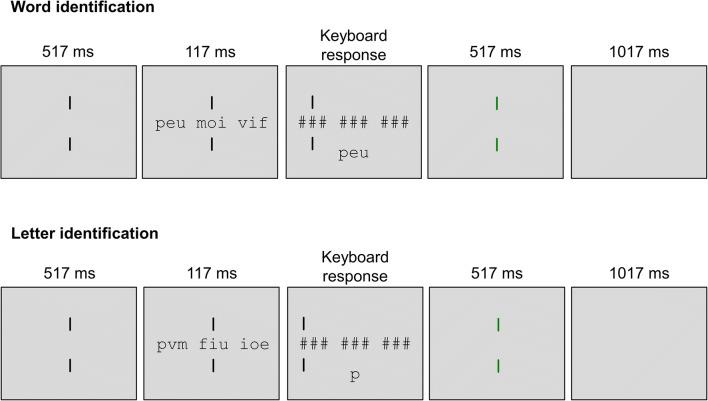
Schematic representation of the experimental procedure for the word- and letter-identification tasks. The first row exemplifies the sequence of events of one trial of the word-identification task requiring to report the first word. The second row exemplifies the sequence of events of one trial of the letter-identification task requiring to report the first letter of the first nonword. Stimuli are not to scale

Before each task (word and letter identification), participants read the instructions and were then administered nine practice trials. Each trial began with a fixation display consisting of two vertical bars positioned at the center of the screen, one above and one below fixation. Participants were instructed to fixate between the two bars. After 517 ms, the target string was displayed for 117 ms. Immediately after, a backward mask was displayed, consisting of a string of nine hash tags (positioned in prior letter locations) with two vertical bars serving as post-cue for the to-be-identified targets. In the word-identification task, the cues appeared one above and one below the middle character of the corresponding word. In the letter-identification task, the two bars appeared one above and one below the to-be-reported letter. Participants were instructed to type the target on the computer keyboard, and their response was echoed on the screen, in uppercase, below the string of hash marks. Self-corrections and editing were allowed via the backspace key. Upon pressing the enter key, the fixation display was restored, and feedback was given by a change in the color of the vertical bars (green = correct response; red = error). The feedback remained on the screen for 517 ms. A blank screen was then presented for 1,017 ms, and served as the inter-trial interval. The procedures for the word- and the letter-identification task are schematically represented in Fig. 1.

For each participant, the word-identification task consisted of 90 trials, and the letter-identification task of 270 trials. Participants could take self-terminated breaks after 45 trials. The order of administration of the two tasks was counterbalanced across participants. The experiment lasted approximately 35 min.

## Results

Response accuracy was analyzed with generalized linear mixed effects models in R (R Core Team, 2020) using the *lme4* package version 4_1.1-21 (Bates, Maechler, Bolker, & Walker, 2015) and the *afex* package version 28.1 (Singman, Bolker, Westfal, Aust, & Ben-Shachar, 2021). For the random effects, we attempted to fit the structure of maximal complexity (Barr, Levy, Scheepers, & Tily, 2013), including by-participants and by-items random intercepts, as well as random slopes for all the different fixed terms (including interactions amongst them and correlations with the intercepts). When these models failed to converge (due to over-parameterization; e.g., Bates, Kliegl, Vasishth, & Baayen, 2018; Matuschek, Kliegl, Vasishth, Baayen, & Bates, 2017) our strategy was to progressively simplify the random-effect structure by: (1) removing correlations (i.e., fitting zero-correlation models), (2) removing random slopes associated with higher order terms (i.e., interactions), and (3) removing slopes with the smallest amount of variance (often corresponding to 0-variance random effects).

The significance of fixed effects was assessed by comparing alternative models in which the fixed effect under examination was either present or absent. Fixed terms were considered to be significant when their inclusion determined an increase in goodness-of-fit as indexed by likelihood ratio tests. In case follow-up pairwise comparisons were conducted, a false-discovery rate adjustment of the p-values was implemented to control for multiple comparisons.

### Word-identification task

**Fig. 2:**
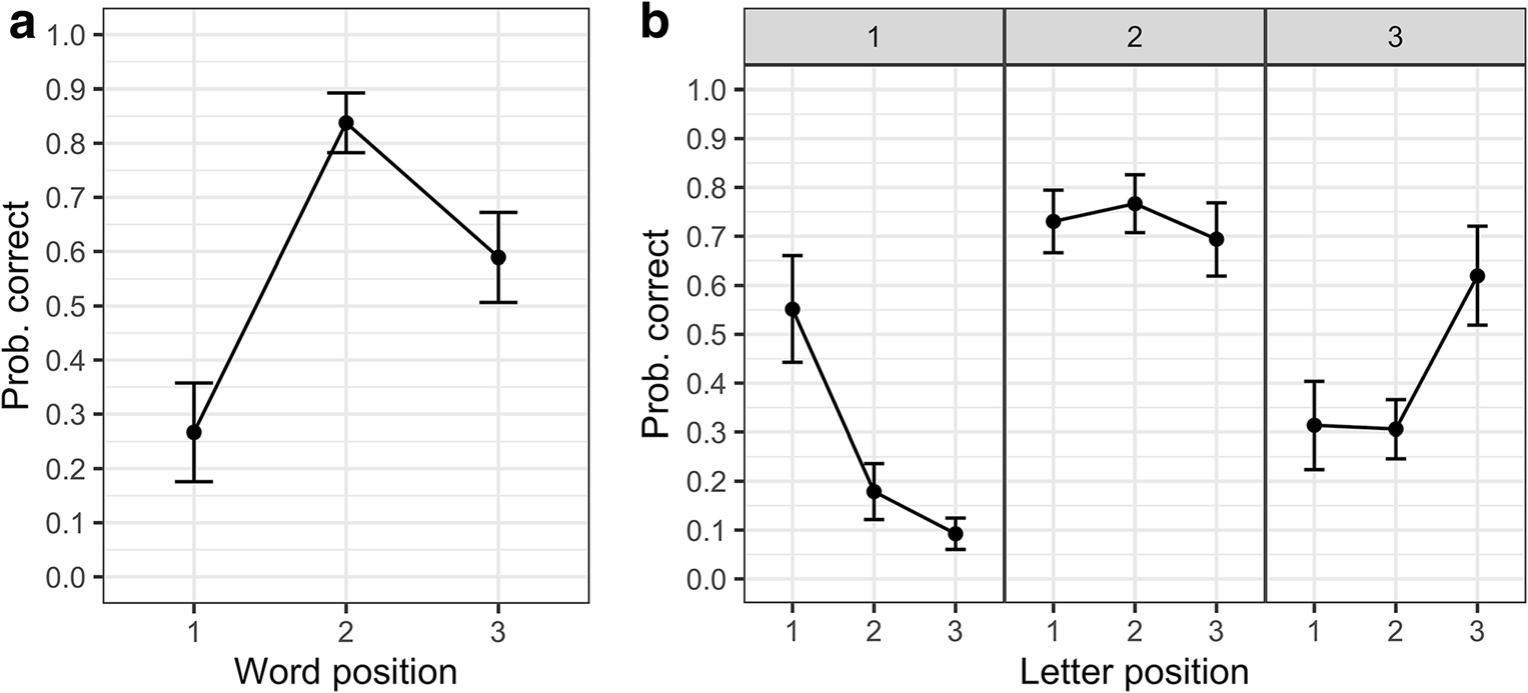
Results of the word- and letter-identification tasks. (**a**): Probability of correct responses to word targets as a function of their position in the sequence. Error bars represent 95% confidence intervals. (**b**): Probability of correct responses in the letter-identification task as a function of letter-in-nonword position (x-axis) and nonword position (panels identified by the labels on top). Error bars represent 95% confidence intervals

The effect of the factor word position (1–3) was significant, *χ*^*2*^ (2) = 35.25, *p* < .001. The random effect structure retained both by-participants and by-items random intercepts and random slopes for the factor word position (and correlations; specification of the model in R: accuracy ~ word position + (1+word position | participant) + (1+word position | item)). Pairwise comparisons revealed a higher chance of correct identification for the word presented at fixation (i.e., in the second position within the sequence) compared both to the words presented in the first (*Estimate* = 3.40, *SE* = 0.40, *z* = 8.42, *p* < .001) or third position (*Estimate* = 1.66, *SE* = 0.34, *z* = 4.93, *p* < .001). Also, the likelihood of a correct response was higher for words presented in the third position, compared to those presented in the first position (*Estimate* = 1.75, *SE* = 0.24, *z* = 7.25, *p* < .001). Mean proportions of correctly identified words as a function of their position within the sequence are presented in Fig. 2 (panel a).

### Letter-identification task

For the letter-identification task there was a significant interaction between nonword position and letter-in-nonword position, *χ*^*2*^ (4) = 361.38, *p* < .001. The final model retained by-participants random slopes for nonword position and letter-in-nonword position (note that random slopes could not be modelled for items, as each nonword was uniquely associated with a specific experimental condition) in a zero-correlation model (specification of the model in R: accuracy ~ nonword position X letter-in-nonword position + (word position + letter-in-nonword position || participant) + (1 | item)). As can be seen in Fig. 2 (panel b), the influence of the position of the target letter had a limited impact for the nonword presented at fixation (i.e., second position in the sequence of three nonwords). On the other hand, for the first and the third nonwords, a much greater accuracy was displayed in identification of the first and the last letters, respectively, compared to the other two positions. Pairwise comparisons are detailed in Table 2.

**Table 2:** Parameters of the pairwise comparisons conducted across the three letter positions in a nonword (1, 2, 3) separately for the first, second, and third nonwords in the sequence of three nonwords

Letters compared	Estimated difference	SE	z	p
First nonword				
1 vs. 2	2.12	0.21	10.21	<.001
1 vs. 3	2.99	0.21	14.04	<.001
2 vs. 3	0.87	0.25	3.52	<.001
Second nonword				
1 vs. 2	-0.19	0.20	-0.94	.39
1 vs. 3	0.21	0.18	1.18	.31
2 vs. 3	0.40	0.21	1.88	.09
Third nonword				
1 vs. 2	0.00	0.20	0.01	.99
1 vs. 3	-1.65	0.18	-9.19	<.001
2 vs. 3	-1.65	0.21	-7.81	<.001

Additionally, we selectively tested the effect of nonword position. This factor determined an increased goodness-of-fit compared to a null model including only random intercepts, *χ*^*2*^ (2) = 534.34, *p* < .001. Also, a model including the factors nonword position and letter-in-nonword position in additive terms displayed a significant increase of explained variance compared to a model featuring only the fixed effect of letter-in-nonword, *χ*^*2*^ (2) = 536.94, *p* < .001. For the final model, we also fitted the by-participants and by-items random slopes for the factor of nonword position (specification of the model in R: accuracy ~ nonword position + (1 + nonword position | participant) + (1 + nonword position | item)). Pairwise comparisons conducted on this model indicated that accuracy for nonword position 1 was significantly lower compared to both nonword position 2 (*Estimate* = -2.77, *SE* = 0.28, *z* = -10.04, *p* < .001) and nonword position 3 (*Estimate* = -1.05, *SE* = 0.20, *z* = -5.34, *p* < .001). Further, accuracy was significantly better for nonword position 2 compared to position 3 (*Estimate* = 1.72, *SE* = 0.24, *z* = 7.27, *p* < .001).

### Relationship between letter and word identification

**Fig. 3:**
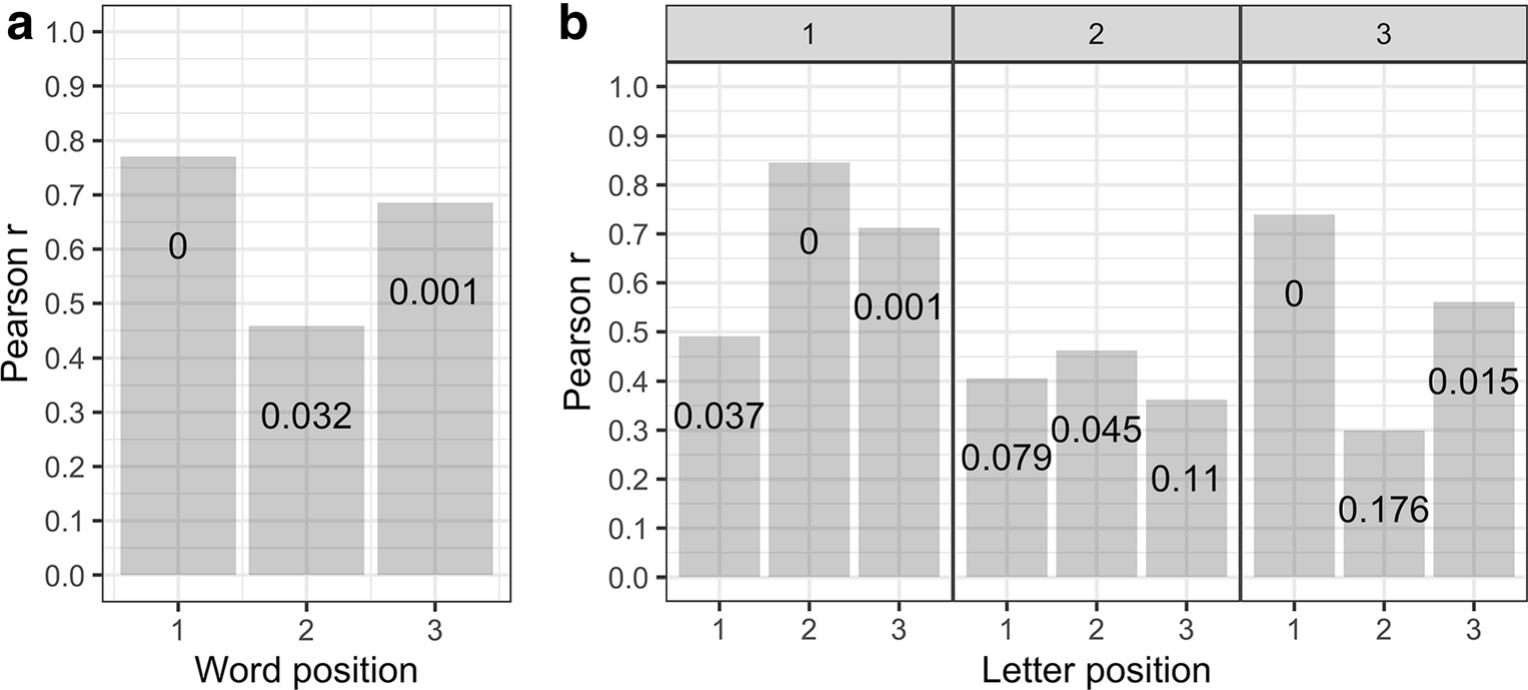
Results of the correlation tests. (**a**) Correlations between word- and letter-identification (collapsed across letter positions within each nonword at a given position) performance as a function of word/nonword position (x-axis). (**b**) Correlations between word- and letter-identification performance as a function of letter-in-nonword position (x-axis) and word position (panels identified by the labels on top). Gray bars represent Pearson r correlation coefficients and corresponding p-values (false discovery rate correction applied) are reported within each bar

First, we explored the correlations between letter- and word-identification accuracies. Specifically, separately for each word position, we tested the correlation between the proportion of accurate responses in the word-identification task and the proportion of correctly identified letters across all letter positions. Results are summarized in Fig. 3 (panel a). Correlations were particularly strong for word positions 1 and 3.

Next, for each word position, we also computed the correlations between the proportion of correctly identified words and the proportion of accurate identification of the corresponding constituent letters in the letter-identification task. Results are summarized in Fig. 3 (panel b). Correlations were rather modest for the central word, and only barely significant for the central letter. In contrast, these correlations are much more substantial for the first and the last words. In these cases, identification of the first and last letter seems to be especially important for accurate word identification.

Finally, we further investigated the relationship between letter and word identification by assessing the extent to which the identification of each constituent letter predicts word identification. For each word, we coded whether the corresponding first, second, or third letter was correctly identified in the letter-identification task. Letter-identification accuracies at different letter positions were then used as separate predictors of the word-identification accuracy within a generalized linear mixed model. Results suggest that only the identification of either the first (*Estimate* = 0.60, *SE* = 0.14, *z* = 4.33, *p* < .001) or the third letter (*Estimate* = 0.40, *SE* = 0.15, *z* = 2.74, *p* = .006) predicts word-identification accuracy (note that the final model retained random slopes for word position; specification of the model in R: accuracy ~ word position + first letter identification + third letter identification + (1+word position | participant) + (1+word position | item)). Participants were significantly more likely to identify the corresponding word when they correctly identified letters at these positions. Importantly, there were no interactions with word position (all *χ*^*2*^*s* < 3.45, *ps* > .17), suggesting that the importance of these letters is not inherently tied to the position of the words within the sequence.

## Discussion

In two separate blocks of trials participants either had to identify one word in a sequence of three three-letter words, or one letter out of nine in a sequence of three three-letter nonwords. All sequences were presented briefly (117 ms), centered on fixation, and immediately followed by a backward mask and post-cue to indicate the position for word or letter report. The component letters of the word stimuli in the word-identification block were tested at the same position in the sequence of nonwords in the letter-identification block. The results are straightforward. Word-identification accuracy was highest for foveal words, and higher for words in the right parafovea than words in the left parafovea. Letter identification accuracy followed the same pattern overall and revealed an interesting W-shaped function when comparing performance across all nine letter positions. We first discuss the word and letter identification results before addressing the crucial relation between the two.

### Word identification

**Fig. B1:**
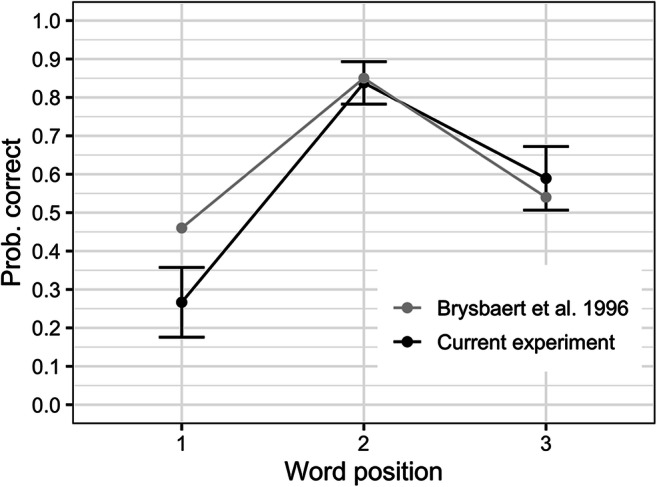
Comparison of the results of the word-identification task in the current experiment versus Brysbaert et al., 1996. Probability of correct responses to word targets as a function of their position in the current experiment (black) and in Brysbaert et al. (1996; grey). Error bars represent 95% confidence intervals. From Brysbaert et al. (1996) we considered their Experiment 2, with three-letter words displayed for 28 ms within comparable positions in terms of eccentricity (labelled as -3, 0, and 3 in the original experiment)

The word identification results revealed a classic right visual field (RVF) advantage (e.g., Brysbaert, Vitu, & Schroyens, 1996; Ducrot & Grainger, 2007; McCann, Folk, & Johnston, 1992; Nicholls & Wood, 1998; Ortells, Tudela, Noguera, & Abad, 1998). In their Experiment 2, Brysbaert et al. (1996) tested isolated three-letter words with fixation either on the word, or to the left or to the right of the word. They found very comparable levels of performance, but with much shorter stimulus durations (28 ms in one condition) compared with the present study. This suggests that the presence of multiple words in the present study was interfering with word identification relative to the single word-presentation procedure of Brysbaert et al. (see Fig. B1 in Appendix B for a comparison of these results and those of the present study). Most important is that the pattern reported by Brysbaert et al. for left parafovea, central, and right parafovea presentations strongly resembles the pattern reported here. This suggests that although having multiple words presented simultaneously causes an overall drop in performance, this does not modify the pattern of differences in foveal and parafoveal word identification. We follow Ducrot and Grainger (2007) in concluding that when words are presented in the parafovea, there is an endogenous attentional bias in the direction of reading.^3^

In line with this conclusion it should be noted that Snell and Grainger (2018) examined effects of flanker position on lexical decisions to central targets in the flanker task, with brief (150-ms) simultaneous presentation of target and flankers. Flankers could be the same words or different words as the central target and could be repeated to the right and left, or uniquely to the right or to the left. Snell and Grainger found stronger effects of flanker relatedness for flankers located to the right. This was taken as evidence that when three words are presented simultaneously, as in the present study, there is an endogenous attentional bias towards the right that causes the greater effects of flanker relatedness for rightward flankers.

Moreover, in the present study we found that the RVF advantage, computed by subtracting average accuracy scores for word position 3 to the scores in position 1, was greater for word stimuli (.32, 95% CI [.24, .41]) compared with nonword stimuli (.14, 95% CI [.09, .19]), which provides further support for an attentional interpretation of this effect that would be driven by reading experience, and therefore stronger in the presence of more readable stimuli.

### Letter identification

**Fig. B2:**
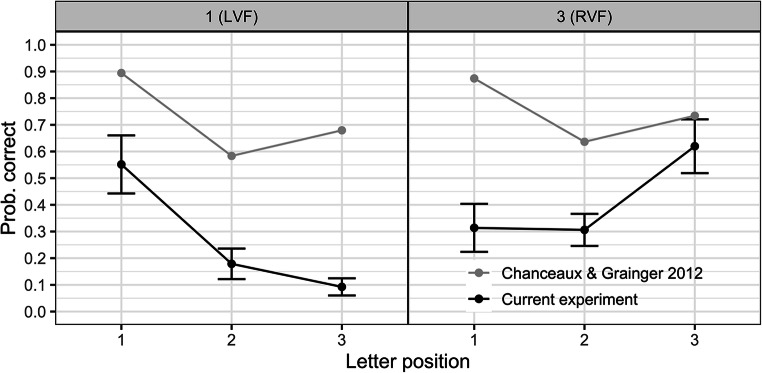
Comparison of the results of the letter-identification task in the current experiment (nonword positions 1 and 3) versus Chanceaux and Grainger (2012). Probability of correct responses to letter targets as a function of their position in the current experiment (black) and in Chanceaux and Grainger (2012). Error bars represent 95% confidence intervals. From Chanceaux and Grainger (2012) we have considered data from Experiment 1 (see their Fig. 3), and averaged accuracy across letter positions 2, 3, and 4 within their five-letter stimuli in order to draw a comparison with our three-letter stimuli. Thus, letter positions 1, 2, and 3 refer to the first letter, three middle letters, and last letter in the five-letter strings tested by Chanceaux and Grainger

The letter identification results revealed an extended W-shaped function that is typically found in foveal letter identification experiments (see Tydgat & Grainger, 2009, for a review). Few studies have examined letter identification in the parafovea using the post-cued single letter report procedure of the present study (see, e.g., Legge, Mansfield, & Chung, 2001, for experiments using a trigram report procedure). Perhaps the most similar parafoveal letter-identification experiments are the ones reported by Chanceaux and Grainger (2012). In that study, strings of five consonants were presented either to the right or to the left of fixation, and a single position among the ten possible positions was post-cued for report, which was a two-alternative forced-choice task in that study. Chanceaux and Grainger also found a distinct W-shaped function spanning the two visual fields, with best performance at the two outer positions and the two positions closest to fixation (for a comparison with the results of our current experiment, see Fig. B2 in the Appendix B). There are two notable differences in the pattern reported by Chanceaux and Grainger (2012). First, there is no RVF advantage, which suggests that exogenous attention, acting when letters strings are presented either to the right or to the left, is cancelling the endogenous bias operating in the conditions tested in the present study. Second, there is improved performance for the innermost letters (closest to fixation), which did not arise in the present study, most likely due to the crowding imposed by the three central letters.^4^

As summarized by Grainger et al. (2016), three factors determine the efficiency of letter identification in letter strings: acuity, crowding, and attention. The combination of these three factors provides a good account of the present letter-identification results. Acuity accounts for the highest accuracy at the three central positions. Crowding accounts for the fact that the next most accurate positions were the two outer positions. Attention accounts for the greater performance in the RVF compared with the LVF. However, there is one aspect of the differences across the RVF and LVF that merits further consideration. This is the fact that the superior performance in the RVF is driven mainly by the two innermost positions – positions 1 and 2 for nonword 3 (RVF) compared with positions 2 and 3 for nonword 1 (LVF). Post hoc pairwise comparisons across nonword positions 1 and 3 as a function of letter-in-nonword eccentricity indeed revealed that identification was significantly better for the first letter of nonword 3 compared to the third one of nonword 1 (*Estimate* = 1.75, *SE* = 0.24, *z* = 7.28, *p* < .001), and for the second letter of nonword 3 compared to the second letter of nonword 1 (*Estimate* = 0.89, *SE* = 0.21, *z* = 4.29, *p* < .001). Further, accuracy was numerically higher for the third letter of nonword 3 compared to the first letter of nonword 1, with the difference approaching conventional significance (*Estimate* = 0.41, *SE* = 0.21, *z* = 1.95, *p* = .051).

This specific pattern is predicted by the Modified Receptive Field (MRF) hypothesis, first proposed by Tydgat and Grainger (2009) and tested and developed in Chanceaux and Grainger (Chanceaux & Grainger, 2012; see also Chanceaux & Grainger, 2013, for a formal analysis, and Chanceaux, Mathôt, & Grainger, 2013, for further tests). The general idea is that crowding effects for letter strings differ in the two visual fields due to an adaptation of receptive fields for letter stimuli during learning to read in order to prioritize processing of initial letters. In order to reduce crowding for initial letters, and maintaining a constant surface area for the receptive fields, it is hypothesized that the oval shape of receptive fields for letters in the LVF are more elongated towards the left and therefore extend less to the right. This predicts that for letters falling in the LVF, the first letter benefits from reduced crowding whereas the following letters suffer from increased crowding compared with letters falling in the RVF at the same eccentricities.

### Letter and word identification

Remember that a key aspect of the design of the present study is that each component letter of a given target word tested at a given position in a sequence of three words was tested at the exact same position in the context of a sequence of three nonwords. In other words, if DOG was tested at the first position in the sequence of words, then D was tested as the first letter in a nonword such as DEJ presented as the first nonword in a sequence of three nonwords, O tested as the second letter in a nonword such as PON, etc. This allowed us to estimate the extent to which word identification accuracy was determined by accuracy of identification of the word’s component letters.

The correlation analyses revealed a strong relation between average letter-identification accuracy in nonwords at a given position and word-identification accuracy at that position. The correlations were highest for the first and third positions (see Fig. 3, panel a). When we break down these correlations per letter-in-nonword position (Fig. 3, panel b), and we focus on nonword/word positions 1 and 3 where the correlations are highest, we note one particularly interesting pattern. In the RVF, the strongest correlation is obtained for letter position 1, while in the LVF, position 1 generates the lowest correlation. This pattern can be explained by the fact that the first letter is the most important letter for word identification, plus the fact that this letter is highly visible in the LVF and much less so in the RVF. In other words, the importance of the first letter for word identification would be modulated by differences in the visibility of each of the letters in the word, with identification of the first letter being all the more important when its visibility is relatively low compared with the other letters in the word. As can be seen in Fig. 2, panel b, identification accuracy of the first letter was much greater compared with letter positions 2 and 3 in the LVF, whereas it was the final (third) letter that was most visible in the RVF. This relation between relative letter visibility and letter-word identification correlations held to a lesser extent for the final position, and not at all for the central position.

Overall, the correlation results suggest that participants who were good at identifying letters in nonwords at a given position were also good at identifying words composed of the same letters at that position. Crucially, the impact of relative letter visibility suggests that it is not just a common underlying factor, such as the ability to identify any kind of visual object in the parafovea, that is driving the correlations. Furthermore, the position-specific correlations provide further evidence for the crucial role played by initial letters in word identification (e.g., Aschenbrenner et al., 2017; Chanceaux & Grainger, 2012; Grainger et al., 2010; Jayawardena & Winskel, 2016; Johnson & Eisler, 2012; Scaltritti et al., 2018; Scaltritti & Balota, 2013; Tydgat & Grainger, 2009; Winskel, Perea, & Peart, 2014; Winskel, Ratitamkul, & Perea, 2018).

## Conclusions

In the present study we measured letter-identification and word-identification accuracy in the fovea and the parafovea while applying testing conditions that were designed to make the two measures as comparable as possible. The correlation analyses revealed a strong relation between the two, hence providing support for models in which word-identification accuracy is principally determined by letter-identification accuracy (e.g., Legge et al., 2001; Snell et al., 2018).

## Figures and Tables

**Fig. A1:**
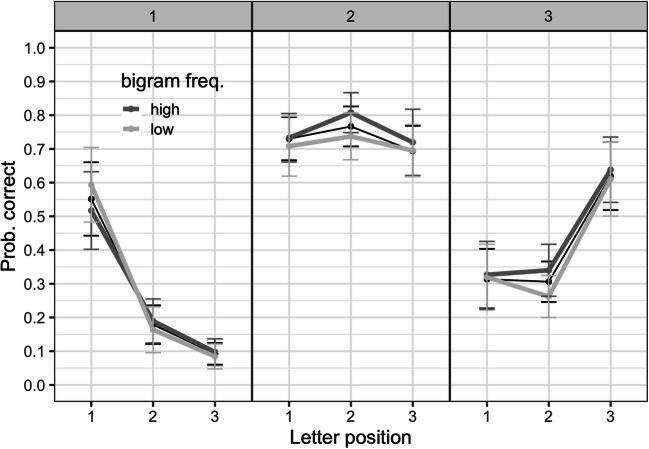
Impact of bigram frequency on the letter-identification task. Mean probability of correct response in the letter-identification task as a function of letter position (x-axis), nonword position (panels, labeled on top), and bigram frequency (high = dark gray; low = light gray; overall = black). Error bars represent 95% CI

## Appendix A

### Control analyses of the letter-identification task considering bigram frequency

As noted by an anonymous reviewer, nonwords in the experiment varied in terms of similarity to word-structures, with nonword strings being potentially either very similar to real words or orthographically illegal. Given the well-known word and pseudoword superiority effects, it is thus conceivable that our letter-identification task may have been influenced by these variations in orthographic structure.

To shed light on this issue, we performed additional analyses considering the impact of bigram frequency on the pattern of results for the letter-identification task. Bigram frequency in fact can capture within a continuous variable the degree of similarity of the different nonword strings with the orthographic structure of the lexicon.

Bigram frequency was computed on the Lexique 3 database (New et al., 2004; New & Pallier, 2020) by calculating the frequency of occurrence of each bigram, weighed by the lexical frequency of the words in which it occurred. For each nonword used in the letter-identification task, we then computed its mean bigram frequency by averaging the frequency of its two constituent bigrams. Mean bigram frequency was entered as a fixed effect in subsequent models.

Compared to the model including fixed effects of nonword position, letter position, and their interaction, the addition of the predictor corresponding to bigram frequency failed to increase goodness-of-fit, *χ*^*2*^ (1) = 0.16, *p* = .69. Nonetheless, we exploratively assessed potential interactions between bigram frequency and the two critical factors of nonword and letter position. None of the two-way interactions involving bigram frequency was significant, *χ*^*2*^s < 1.11, *p*s > .57. The three-way interaction (nonword position X letter position X bigram frequency) merely approached conventional significance, *χ*^*2*^ (4) = 8.19, *p* = .08. This indication of an interaction may stem, according to the model’s estimate, from the fact that higher bigram frequency seems to enhance the identification of the third letter compared to the first one (letter position 3 × bigram frequency: *b* = 0.44, *SE* = 0.21, *z* = 2.09, *p* = .04), albeit this would be significantly reduced for nonwords in position 2 compared to position 1 (word position 2 × letter position 3 × bigram frequency: *b* = -0.57, *SE* = 0.26, *z* = -2.14, *p* = .03). Given the marginal interaction and the lack of a bigram-frequency effect, we believe it is safe to conclude that the similarity of the nonwords with real words, possibly due to its random distribution across experimental conditions, played a negligible role in shaping any of the results.

Figure A1 reports the results in the letter-identification task, broken down for nonwords featuring a high versus a low bigram frequency (as determined via median-split) as well as the overall results for the sake of comparison. The pattern is highly similar across all the different sets of nonwords.

## Footnotes

1 We acknowledge here the seminal work of Gordon Legge and colleagues (e.g., Legge et al., 2001) in an attempt to link letter-identification accuracy to reading speed in central and peripheral vision. Our approach differs in terms of methodology (e.g., horizontal as opposed to vertical periphery, and more directly comparable testing conditions for letters and words), but the obtained results are in accordance with those of Legge et al.

2 Following Snell and Grainger (2017) and other related studies, and given our focus on multi-word processing rather than isolated word recognition, we used lowercase fonts, which is the typical format for texts.

3 We note nevertheless, that visual field effects obtained with simultaneously presented words can be modulated by the distance separating these words (e.g., Van der Haegen & Brysbaert, 2011). In the present study we used a single character space between the three stimuli, hence mimicking the conditions of natural reading. In the Van der Haegen and Brysbaert study, the inversion of the RVF advantage to an LVF advantage emerged with two-character spacing between two stimuli centered on fixation, but one-character spacing was not tested.

4 A potential alternative interpretation, proposed by an anonymous reviewer, is that the shorter presentation durations of the present study (117 ms compared with 300 ms in the Chanceaux & Grainger study) forced participants to focus their attention more highly on the central stimulus (see Fernández-López, Marcet, & Perea, 2020, for an alternative to such a mechanism in explaining different patterns in letter-in-string identification). However, we would have expected more focused attention on central stimuli to have benefitted processing of the last letter of nonwords at position 1 and the first letter of nonwords at position 3, rather than hindering identification of letters at these positions.
